# Case Report: Interdisciplinary management of a complex odontoma with a periapical involvement of superior anterior teeth

**DOI:** 10.12688/f1000research.20337.1

**Published:** 2019-08-29

**Authors:** Esteban Isaí Flores Orozco, Amjad Abu Hasna, Moacir Teotonio de Santos Junior, Elan Ignacio Flores Orozco, Renata Falchete Do Prado, Gabriel Rocha Campos, Marcia Carneiro Valera

**Affiliations:** 1 Department of Restorative Dentistry, Endodontic Division, Institute of Science and Technology, São Paulo State University (UNESP), São José dos Campos, São Paulo, 12245-000, Brazil; 2 Department of Science and Technology Applied to Dentistry, Institute of Science and Technology, São Paulo State University (UNESP), São José dos Campos, São Paulo, 12245-000, Brazil; 3 Department of Prosthodontics, Faculty of Dentistry, Autonomous University of Nayarit, Tepic, Nayarit, 63155, Mexico; 4 Department of Social Dentistry and Children's Clinic, Institute of Science and Technology, São Paulo State University (UNESP), São José dos Campos, São Paulo, 12245-000, Brazil; 5 Faculty of Medicine and Dentistry, São Leopoldo Mandic, São Paulo, 01332-000, Brazil

**Keywords:** Complex odontoma, Five mineral oxides, Bioceramics, Computed tomography

## Abstract

This case report aims to describe the management of a complex odontoma with endodontic involvement of surrounding teeth utilizing a new bioceramic cement consisting of five mineral oxides (5MO) as a retro-filling material. The patient presented for routine consultation with slight dental mobility in the antero-superior region. Upon clinical and computed tomography examination, bone rarefaction was observed in the apical region of teeth 11 and 12, external root resorption in the involved teeth and necrotic pulp of tooth 12. Root canal treatment was performed in teeth 11 and 12. Later, local surgical excision of the lesion was carried out, finding a mass with clinical features of complex odontoma, with histopathological examination of the mass confirming this diagnosis. Retro-filling of tooth 12 with 5MO was carried out. No signs and symptoms were observed over twelve-months of follow-up, with bone neoformation observed in the region. Therefore, 5MO appears to be an effective bioceramic cement that has reparative features.

## Introduction

Odontogenic tumors originate from tooth-forming tissues ( Siriwardena *et al.*, 2019), and are divided based on their origin into epithelial odontogenic tumors, kerato-cystic odontogenic tumors, mixed odontogenic tumors, and mesenchymal odontogenic tumors ( Wright & Vered, 2017).

Odontomas are benign odontogenic tumors of mixed origin (epithelial and mesenchymal), composed mainly of mineralized and conjunctive structure including enamel, dentin, cementum and pulp ( Wood & Goaz, 1997). Odontomas are subdivided into compound and complex odontomas; compound odontoma corresponds to small variable structures morphologically similar to teeth, whereas complex odontomas have no anatomical similarity with teeth and present as a structural conglomeration of enamel and dentin ( Wright & Vered, 2017).

Complex odontomas are mostly asymptomatic, are usually diagnosed during the first and second decade of life ( Budnick, 1976), and are more prevalent in the maxilla than in the mandible ( Ahire *et al.*, 2018). They may have a differential radiographic diagnosis with highly calcified lesions like osteoma and adenomatoid odontogenic tumor, among others ( Sun *et al.*, 2015). Odontomas are treated by local surgical excision and the prognosis is favorable without recurrence ( Gervasoni *et al.*, 2017).

Five mineral oxides (5MO) is a new bioceramic cement that’s use has been described in the literature as an apical plug of open apex teeth ( Altaqi, 2014) and a capping material ( Ala Rachi *et al.*, 2014). It is a modified MTA (mineral trioxide aggregate) material that consists of five mineral oxides.

## Case report

### Case presentation and patient information

A Brazilian white 27-year-old female presented for routine examination with a slight dental mobility in teeth 11 and 12 with no history of trauma or previous treatments. Patient clinical history did not present relevant findings.

### Patient assessment

**Figure 1.**
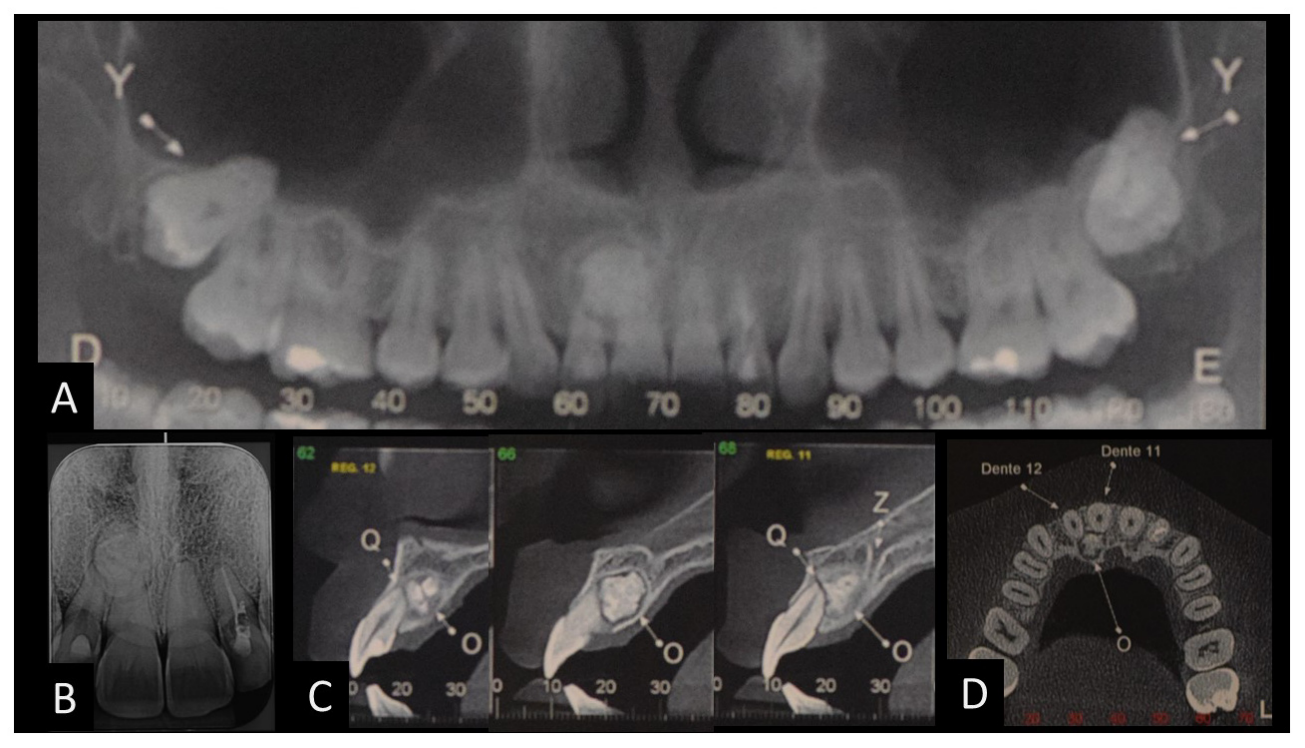
The radiographical diagnosis of complex odontoma. **A**) and **B**) panoramic and periapical radiography showing radiopaque lesion in the upper right incisors region; **C**) tomographic image of the sagittal section showing the relation of the lesion with the apex of the upper right central and lateral incisors; **D**) axial reconstruction of odontoma.

The patient had no symptoms and no history of trauma or previous treatments in this region. Teeth 13 to 23 was tested by pulp vitality test (cold test) carried out by refrigerant gas (Endo Ice, Maquira Dental products industry LTDA–Brazil) and relative isolation using cotton rolls and a dental saliva ejector. The apical region was also tested by digital palpation and perpendicular percussion and was asymptomatic, with no fistula. The depth of its gingival pocket varied between 1–3 mm with various exploring locations and grade I mobility. Panoramic radiographic examination revealed a radiopaque bone-like structure but with a greater density than bone, circumscribed by a thin radiolucent margin in the apical region of teeth 11 and 12 presenting features of complex odontoma ( Figure 1). Cone beam computed tomography (CBCT) was indicated to obtain an accurate diagnosis of the lesion and its relationship with the adjacent teeth. CBCT was fundamental in the surgery planning and management in choosing the most appropriate access, which was palatal access in this case.

### Therapeutic interventions

Firstly, teeth 11 and 12 underwent endodontic treatment due to the negative response to the pulp vitality test, utilizing a Reciproc system R50/0.05 file (VDW, Munich, Germany) and chlorohexidine gluconate gel 2% (Endogel Essential Pharma, Itapetininga, Brazil) as a lubricator and antimicrobial agent during the preparation of the root canals, and then obturating with gutta-percha and Ah Plus sealer (Dentsply, DeTrey GmbH, Konstanz, Germany). No medication was prescribed before or during the treatment. The patient was advised to take acetaminophen (500 mg, maximum four times a day) in case of pain. 

A week later, after tomographic planning, surgical excision of the lesion was carried out under local anesthesia using one anesthetic tube (4% articaine with epinephrine 1: 100,000), with intra-oral access to the mass achieved via intra-sulcular incision of the palatal region from tooth 14 to 24. After detachment of the flap, osteotomy was performed using a surgical carbide drill nº 06 (Angelus Prima Dental Ltda. Londrina / PR, Brazil) under intense irrigation with saline solution.

**Figure 2.**
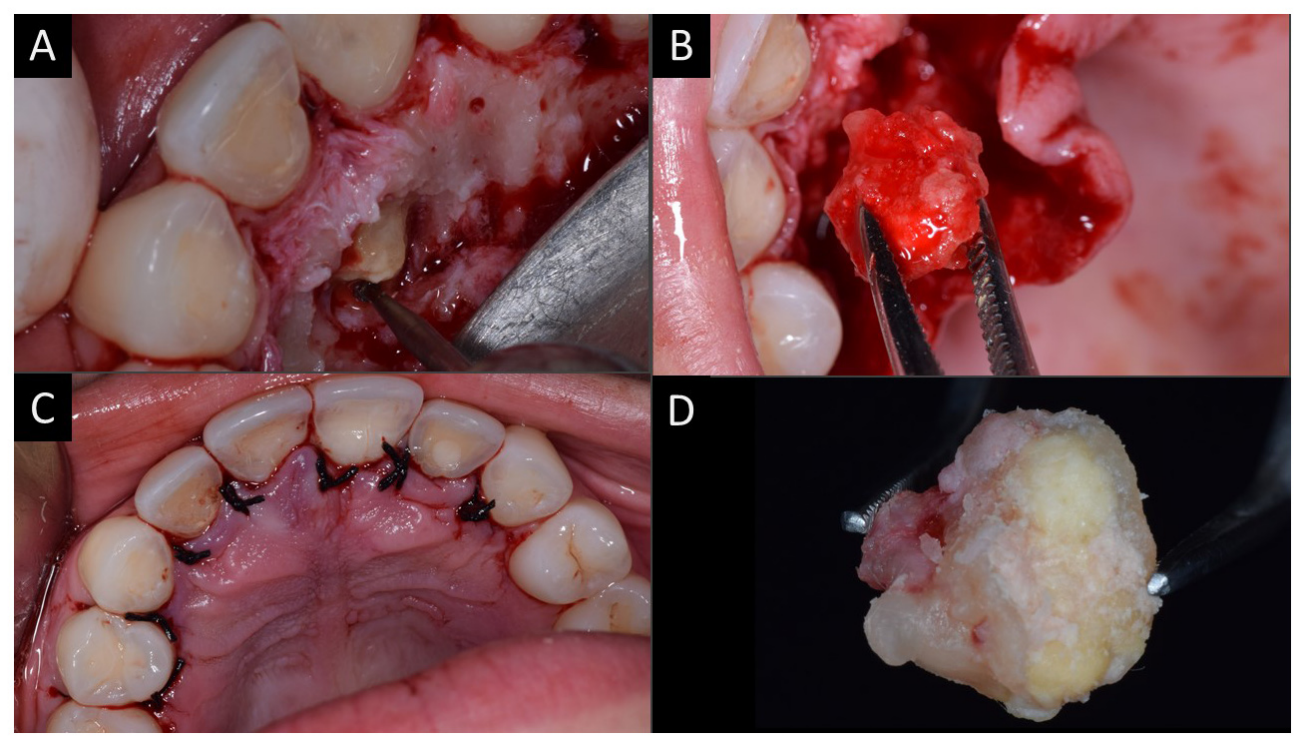
Surgical approach for total excision of the complex odontoma. **A**) Osteotomy; **B**) total excision of the odontoma; **C**) repositioning of flap and intra-sulcular suture; **D**) enucleated complex odontoma.

The enucleated lesion presented as a hyperdense mass located palatal to the roots of teeth 11 and 12, with clinical features of complex odontoma that involved the apical third of the tooth 11. A bone regularization procedure was carried out, followed by cleaning of the surgical cavity by irrigation with saline solution ( Figure 2).

**Figure 3.**
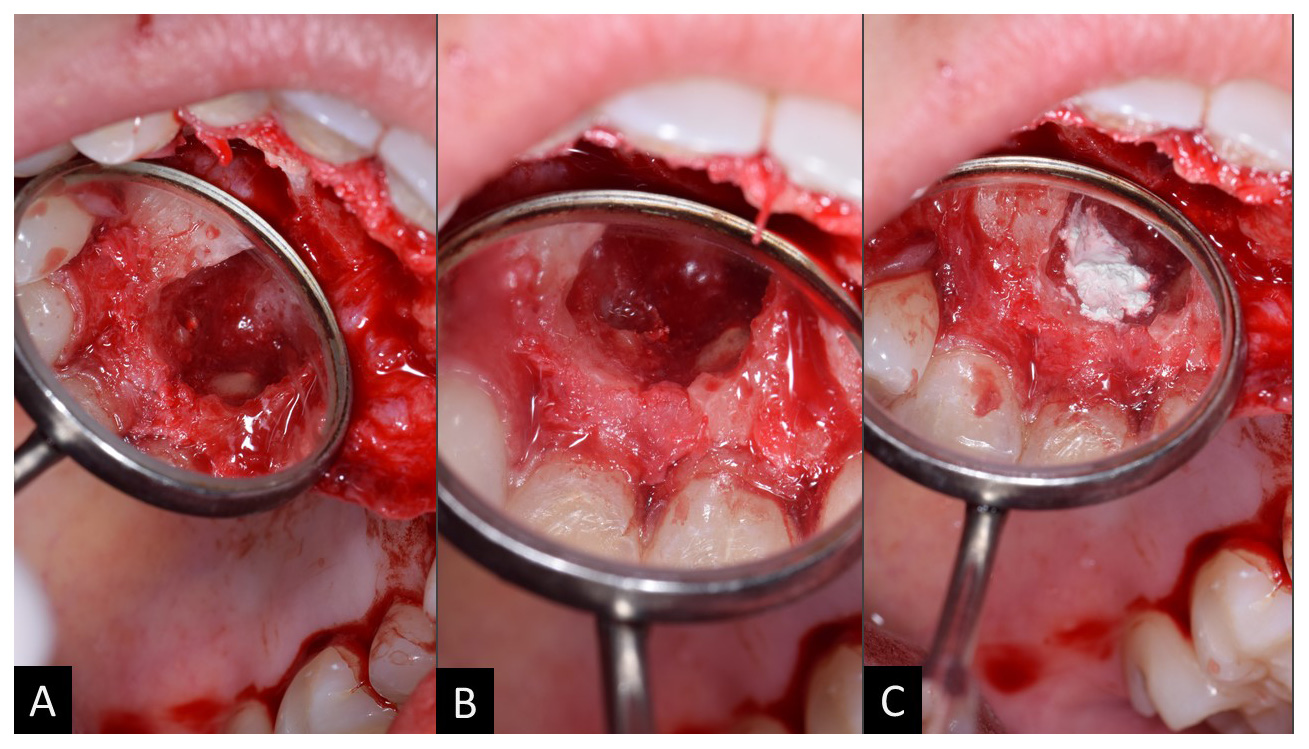
Clinical approach to retro-obturation with five mineral oxides (5MO). **A**) Image of the surgical access after washing and polishing the rigid structures with curette; **B**) creation of a niche in the apical third for des-obturation; **C**) placement of 5MO cement in the retro-obturation.

Afterwards, the hard-dental surfaces were polished with McCall periodontal curette (Fava Metalúrgica São Paulo, SP). Tooth 12 demonstrated external apical resorption; thus, an apicectomy was performed, which was then retro-filled with 5MO (SHAM Dentico, Oman) ( Figure 3). No bone graft was indicated. The flap was repositioned, followed by intra-sulcular suturing with 3-0 silk thread (Procare Xuyi Webest Medical Products Co, Jiangsu China). The suture was removed after seven days and the patient progressed well postoperatively without intercurrences.

### Histopathological assessment

Immediately after sample excision, the mass was immersed in 10% formalin for 48 hours in order to obtain tissue fixation and was sent for histopathological examination under a microscope for differential diagnosis. Routine histological techniques were performed for soft tissues embedding in paraffin. Hard tissues fragments were demineralized in Ethylenediaminetetraacetic acid (EDTA) (TiTriplex® Merk Darmstadt, Germany) for two months, before embedding. The paraffin blocks were cut into sections and slides were subjected to hematoxylin and eosin (HE) staining and observed using an optical microscope. All slides images were obtained using a Pannoramic Scan (3DHISTECH, Budapest, Hungary).

### Follow-up and outcomes

After seven days, no postoperative intercurrences were reported by the patient. Through intra oral evaluation, no hematoma or edema was noticed except for the first two days as the patient mentioned. No exudate was noticed.

Soft tissue sections (correspondent to tumor surrounding tissue) demonstrated fragments of connective tissue composed of dense collagen fibers randomly arranged, with fibroblasts and hyalinization areas. Immersed in bundles of collagen fibers, many basophilic round calcification areas were observed. Inflammatory cells were not present. Odontogenic islands were observed.

**Figure 4.**
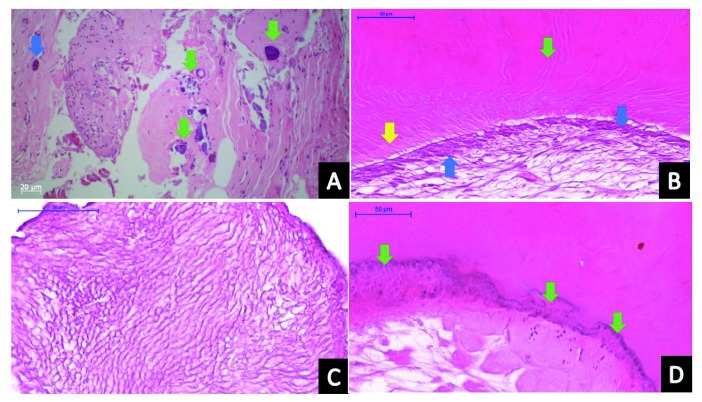
Histopathological analysis of the odontoma. **A**) Connective tissue around the tumor showing bundles of collagen representing mineralized areas (green arrows) and an island of odontogenic epithelium (blue arrow); **B**) pulpal tissue-like area, demonstrating delicate collagen fibers, a columnar layer of odontoblasts (blue arrows) related to the pre-dentin (yellow arrow) and the dentin matrix showing dentinal tubules (green arrow); **C**) enamel matrix; **D**) mineralized tissue similar to cement, presenting basophilic reverse lines (green arrows).

Histologically, the tumor mass was composed of tooth-like structures exhibiting an irregular arrangement. Hard tissue sections presented fragments of a mixture of material composed mainly of immature dentin, enamel matrix, cementum, and pulp tissue. Dentin immature matrix presented irregular tubules; enamel matrix demonstrated a basophilic pattern and barely prismatic spaces from hydroxyapatite loss during the demineralization process; cementum areas were superimposed to dentin, differing from dentin because of its basophilic irregular lines and the presence of cells inside the matrix (cellular cementum); finally, pulp tissue was observed surrounded by dentin matrix, composed of delicate collagen fibers and fibroblasts. In some places, the dentin matrix surface presented columnar cells similar to odontoblasts. Histopathological examination confirmed the diagnosis of complex odontoma ( Figure 4).

**Figure 5.**
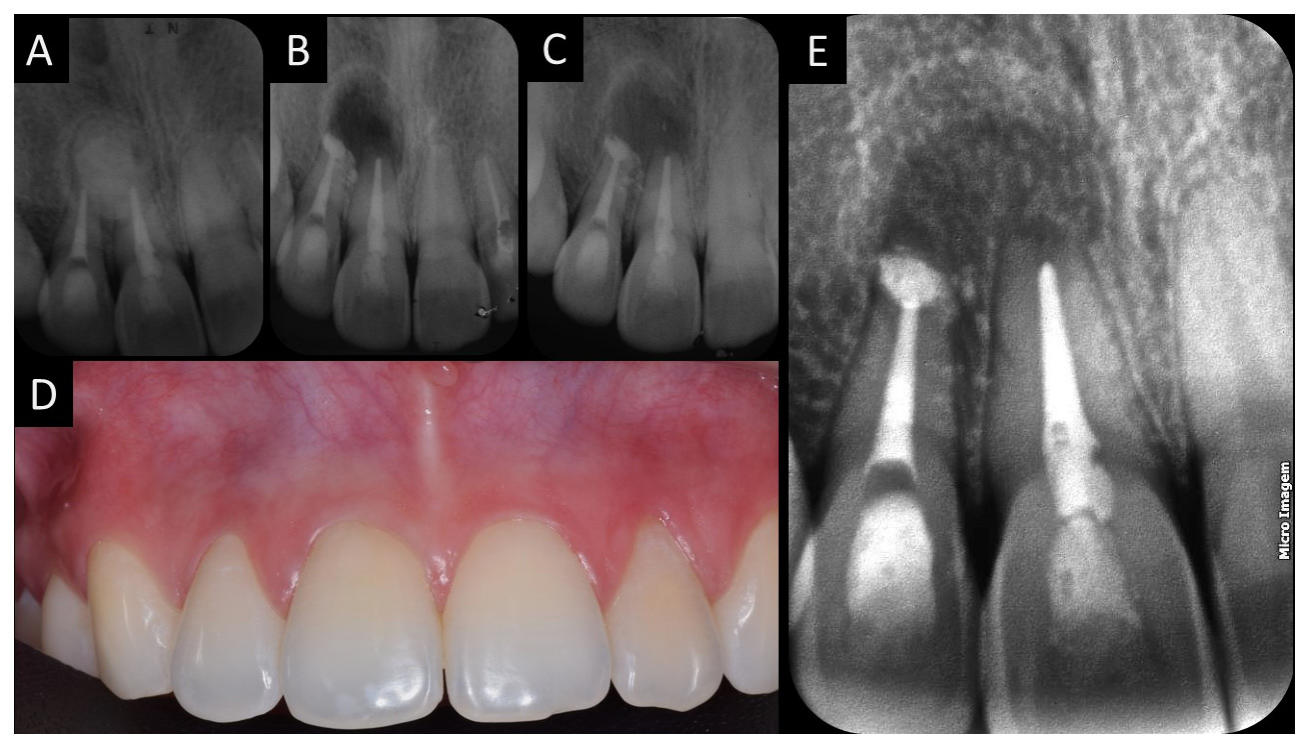
Radiographic preservation. **A**) Periapical radiography of the endodontic treatment before surgery; **B**) radiographic examination after excision of the odontoma and performing the apical retro-obturation of tooth 12; **C**) 30 days after surgery; **D**) and **E**) 12 months after removal of the odontoma.

Three follow-up sessions were performed after 30 days, four months and 12 months. In these sessions, clinical intra oral examination was carried out as well as radiographic examination. Bone neoformation was observed by periapical radiograph in the lesion region at the second follow-up session (after four months) compared to the initial one. The periapical radiograph was taken using a radiographic positioner ( Figure 5).

## Discussion

Odontoma is an anomaly of benign asymptomatic development and slow growth, ( Tomizawa *et al.*, 2005). Its early diagnosis enables management of the tumor at the most opportune moment, avoiding further complications and healthy structures being compromised.

Odontoma is more common between the first and second decade of life. Thus, some studies have focused on diagnosis in pediatric patients ( Katz, 1989; Tomizawa *et al.*, 2005). In a Brazilian community, de Medeiros *et al.* (2018) evaluated the frequency of odontogenic tumors over a period of 22 years and verified that odontoma is the most common tumor and is more frequent in the second and third decade of life. This is in agreement with the case described here, as the patient was diagnosed at 27-years-old, without compromising the normal eruption of the permanent teeth. Another similar study conducted over 26 years found odontoma was the 4 ^th^ most frequently detected tumor in an African community, with greater prevalence in maxilla than in mandible, and in anterior teeth than in premolars and molars ( Mamabolo *et al.*, 2011), as found in the present case report.

A further study conducted over 12 years revealed that the prevalence of odontoma is relatively low in African countries in comparison to more developed countries ( Aregbesola *et al.*, 2018). Similar results of a low prevalence were found in a Brazilian community, in which odontoma was more frequent in mandible than in maxilla and in females than males ( Lima-Verde-Osterne *et al.*, 2017). Furthermore, similar results were found in another study, reporting a low prevalence of odontoma in a 10 year study in an Indian community, with the majority of cases occurring in male patients ( Mehngi *et al.*, 2018). 

The use of CBCT was fundamental in this case report; since the indicated treatment of odontoma is the surgical enucleation, CBCT helped in the planning and identification of calcified structures ( Sun *et al.*, 2015). CBCT provides a three-dimensional view of the continuity, size and volume of lesions for surgical consideration, at a relatively low radiation dose and high resolution ( Favia *et al.*, 2000; Sun *et al.*, 2015).

Histopathological analysis is also important in cases of odontoma. The sample should be carefully examined under the microscope to confirm the differential diagnosis.

Many bioceramics and MTA-like cements induce osteogenic differentiation and stimulate the repair of lesions ( Margunato *et al.*, 2015); in this case report, 5MO was effective in promoting apical sealing, which contributed to the osteogenic differentiation and the repair of the lesion. The performed intervention was successful, due to the absence of pathognomonic signs and symptoms, showing a biocompatibility in the presence of 5MO cement in the apical region.

MTA as retro-filling material is better than amalgam and purely gutta-percha in endodontic surgery ( Tang *et al.*, 2010). In this case report, the 5MO cement used was effective in a case of periapical involvement and acted as the MTA as it is an MTA-like material.

The surgical planning using CBCT analysis, the parendodontic intervention through apical retro-filling with the 5MO, and the histopathological analysis of the sample described here reinforces the best pre-established protocol of the treatment ( Gervasoni *et al.*, 2017).

## Data availability

All data underlying the results are available as part of the article and no additional source data are required.

### Consent

Written informed consent for publication of their clinical details and clinical images was obtained from the patient.
