# Supplementary material for: Case Report: Interdisciplinary management of a complex odontoma with a periapical involvement of superior anterior teeth
Source: F1000Res. 2019 Aug 29;8:1531. [Version 1] doi: 10.12688/f1000research.20337.1 (PMC6880268; doi:10.12688/f1000research.20337.1)
Supplement: Peer review history

# Peer review: Reviewer response for version 1

**DOI:** 10.5256/f1000research.22347.r56260

**Published:** 2019-11-21

**Authors:** Fabio da Silva Matuda

**Affiliations:** 1 Periodontics, University of Paraíba Valley (Univap), São José dos Campos, Brazil

The paper is suitable for indexing, but I suggest that was necessary the bioethics committee approval number. The work description is very detailed and the applied exams allow elucidation of the case studied. The pictures are of excellent quality and clearly demonstrate the clinical technique used to remove the lesion.

I confirm that I have read this submission and believe that I have an appropriate level of expertise to confirm that it is of an acceptable scientific standard.

# Peer review: Reviewer response for version 1

**DOI:** 10.5256/f1000research.22347.r53151

**Published:** 2019-09-30

**Authors:** Talal Al-Nahlawi

**Affiliations:** 1 Department of Endodontics and Operative Dentistry, Syrian Private University (SPU), Damascus, Syria

This case report describes a proposal of the use of a new bioceramic cement which is indicated as a retrofitting or a perforation repair material in Endodontics.

 The abstract is fully comprehensive.

 The introduction presented a brief explanation of odontoma and the classification of odontoma (complex and compound odontoma) , in addition to a brief phrase the new bioceramic cement of five minerals oxide 5MO. However, more details should be provided about this new cement, such as its composition and its clinical applications.

 The case report part was well explained, however, more control period is indicated for 2, and 5 years until the full healing is obtained, and make sure about the effectivity of this bioceramic cement.

 The discussion section was good as well. As a suggestion for the authors, the 5MO cement should be compared in the discussion with the similar bioceramic cements like MTA and its new versions like MTA HP and others, this should be more discussed.

I confirm that I have read this submission and believe that I have an appropriate level of expertise to confirm that it is of an acceptable scientific standard.
